# Nilotinib-induced metabolic dysfunction: insights from a translational study using in vitro adipocyte models and patient cohorts

**DOI:** 10.1038/s41375-018-0337-0

**Published:** 2019-01-28

**Authors:** Soban Sadiq, Euan Owen, Terry Foster, Katy Knight, Lihui Wang, Munir Pirmohamed, Richard E. Clark, Sudeep Pushpakom

**Affiliations:** 1 Molecular and Clinical Pharmacology, University of Liverpool, Liverpool, United Kingdom; 2 Royal Liverpool and Broadgreen University Hospitals NHS Trust, Liverpool, United Kingdom; 3 Institute of Translational Medicine, University of Liverpool, Liverpool, United Kingdom

**Keywords:** Chronic myeloid leukaemia, Translational research

## To the Editor:

Nilotinib, a second-generation tyrosine kinase inhibitor (TKI), has been described to be a superior drug in the frontline treatment of patients with Philadelphia chromosome–positive (Ph+) chronic myeloid leukemia (CML) [1]. However, with more mature follow-up, it has become clear that nilotinib is associated with impaired glucose and lipid metabolism [2–4] and an excess in arterial thrombotic events in comparison to imatinib [5]. The 5-year safety update of the ENESTnd trial [3] provided further confirmation; it reported significant elevations in fasting glucose and serum lipids and an increased incidence of cardiovascular events in nilotinib-treated patients as opposed to imatinib [3].

Adipose tissue is an important determinant of whole body glucose and lipid homeostasis [6], and adipocyte dysregulation is known to result in various metabolic abnormalities [7]. Accumulation of drugs in adipose tissue could result in adipocyte toxicity; we have shown that anti-HIV drugs cause adipocyte toxicity leading to insulin resistance and the development of cardiometabolic disease in HIV-positive individuals [8]. We hypothesised that nilotinib could cause adipocyte toxicity leading to various metabolic adverse effects in CML patients; here we have undertaken a translational study using in vitro–in vivo models to characterise this. We have also tested telmisartan, an angiotensin receptor blocker (ARB) and antihypertensive with beneficial metabolic effects [9], as a potential therapeutic strategy to reduce nilotinib-induced metabolic toxicity in vitro.

A chronic in vitro toxicity model as previously described [8], consisting of 3T3-F442A murine preadipocyte cells, was used to investigate the effect of nilotinib and imatinib on adipocytes. Briefly, differentiating adipocytes were incubated with either nilotinib (with or without telmisartan) or imatinib 48 h post-initiation of differentiation, and drug treatment was continued every 48 hours over a period of 10 days to mimic the chronic dosing schedule in CML patients. Nilotinib (1–4 μM) and imatinib (5 μM) were used within their therapeutic range; given the lipophilicity of nilotinib, we also assessed a hypothetical higher nilotinib concentration (20 μM) assuming adipose tissue accumulation following chronic drug treatment. Lopinavir, an anti-HIV drug known to cause adipocyte toxicity and metabolic disturbances [8], was used as positive control. We have only investigated these two TKIs in the current study. Statistical analyses were conducted by one-way ANOVA with Dunnett’s Test. All in vitro experiments were repeated three times in triplicate. A *p* value ≤ 0.05 was considered significant.

**Fig. 1:**
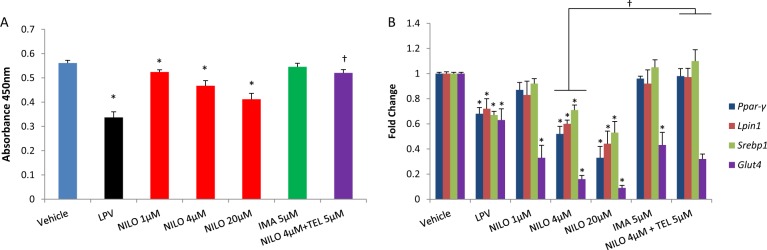
Effect of nilotinib (with or without telmisartan) and imatinib on adipocyte lipid accumulation and mRNA expression of *Pparγ*, *Lpin1, Srebp1* and *Glut4* in differentiating 3T3-F442A adipocytes. 1**A** Lipid droplets were stained with Oil Red O on day 10 following treatment with respective drugs/vehicle. Lipid bound Oil Red O was extracted with isopropyl alcohol and absorbance was measured at 450 nm. 1**B** Gene expression of *Pparγ*, *Lpin1, Srebp1* and *Glut4* were assessed by real-time PCR using Taqman assays-on-demand gene expression assays (Life Technologies) on a 7900HT Fast Real-Time PCR system (Life Technologies). *Hprt* was used as an endogenous control. The mRNA expression was calculated using the comparative Ct method according to the manufacturer’s protocol and the fold change for the gene of interest was expressed as 2^^-(∆∆CT)^. Telmisartan was co-incubated with only one concentration of nilotinib (4 µM). Lopinavir (LPV), an anti-HIV drug, was used as a positive control. All experiments were repeated three times in triplicate. Statistical analyses were conducted by one-way ANOVA with Dunnett’s Test. Data represent Mean ± SD; *p* ≤ 0.05. *Vehicle vs NILO/LPV/IMA; ^†^NILO4µM vs NILO4µM + TEL5µM. NILO: nilotinib, IMA: imatinib, TEL: telmisartan, LPV: lopinavir, *Hprt:* Hypoxanthinephosphoribosyltransferase

We investigated whether nilotinib and/or imatinib (0.01–100 µM) caused cytotoxicity in both undifferentiated and differentiating adipocytes using the MTT assay. Neither nilotinib nor imatinib reduced cell viability in these cell types at clinically relevant concentrations (Supplementary Figure 1). We hypothesised that nilotinib may interfere with adipocyte lipid accumulation and alter mRNA levels of key adipogenic regulatory genes (*Pparγ*, *Lpin1, Srebp1*). Lipid accumulation in differentiated adipocytes was assessed on day 10 using Oil Red O staining [8] and gene expression was assessed by Real-Time PCR using Taqman assays (Life Technologies). Nilotinib (4 µM: 0.46 absorbance units±0.02, *p* = 0.001), but not imatinib, caused dose-dependent reduction in adipocyte lipid accumulation when compared with the vehicle (0.56 ± 0.01) (Fig. 1A; also see Supplementary Figure 2). Reduced lipid droplet formation observed with nilotinib may suggest the inability of adipose tissue to store lipids; this will result in the ectopic accumulation of fat in the liver and skeletal muscle leading to the development of insulin resistance [10]. Nilotinib, but not imatinib, also resulted in dose-dependent downregulation of all three adipogenic regulatory genes, with the effect evident at therapeutic concentrations (4 µM nilotinib: *Ppar-γ*: 48% downregulation, *Lpin1*: 40% downregulation, *Srebp1*: 29% downregulation; all *p* < 0.05; Fig. 1B). *PPARγ* is a master regulator of adipogenesis and mediates adipogenic gene expression and insulin sensitivity [11]; *lipin1*, a gene that encodes a magnesium-ion-dependent phosphatidic acid phosphohydrolase enzyme, is involved in triglyceride synthesis [12]; *SREBP1* plays a role in cholesterol homeostasis [13]. We then assessed the effect of these two TKIs on *Glut4*, the principal glucose transporter in the adipocyte; both nilotinib (*p* = 0.01), and to a lesser extent imatinib (*p* = 0.02), significantly downregulated *Glut4* mRNA expression in differentiating adipocytes (Fig. 1B). Downregulation of *Glut4* by nilotinib could result in reduced glucose uptake into the adipocyte and may lead to insulin resistance observed in CML patients. Downregulation of *Glut4* by imatinib, a drug that has been consistently suggested to improve insulin sensitivity in CML patients [14], is interesting; this suggests the need to assess other mechanisms involved in the regulation of whole body insulin sensitivity, such as the role of liver and skeletal muscle. We then assessed whether telmisartan can reverse nilotinib-induced adipocyte toxicity; co-incubation of telmisartan (5 µM) with 4 µM nilotinib resulted in significant reversal of nilotinib-mediated inhibition of adipocyte lipid accumulation (NILO + TEL: 0.52 ± 0.01, in comparison to NILO 4 µM: 0.46 ± 0.02, *p* = 0.01; Fig. 1A) and adipogenic mRNA downregulation (*p* = 0.02; Fig. 1B).

**Fig. 2:**
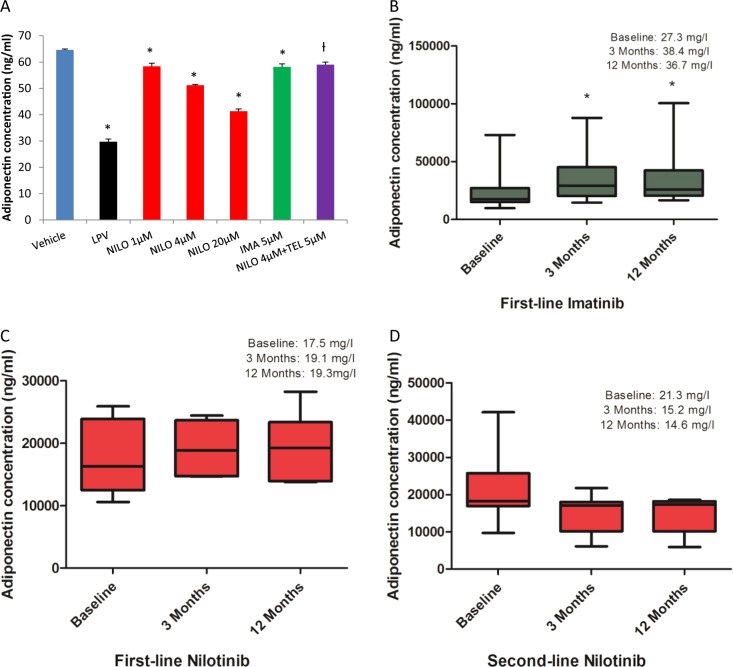
Effect of nilotinib and imatinib on adiponectin in vitro and in vivo. Effect of nilotinib (with and without telmisartan) and imatinib on secreted adiponectin in differentiating 3T3-F442A adipocytes (**A**); plasma adiponectin levels at baseline, 3 months and 12 months in CML patients treated with imatinib (**B**); first-line nilotinib (**C**) and second-line nilotinib (**D**). Telmisartan was co-incubated with only one concentration of nilotinib (4 µM). Lopinavir (LPV), an anti-HIV drug, was used as a positive control in vitro. All in vitro experiments were repeated three times in triplicate. One-way ANOVA with Dunnett’s Test was used for in vitro statistical analysis; Repeated measures ANOVA with Dunnett’s Test was used to compare plasma adiponectin levels at different time points in CML patients. Data represent Mean ± SD; *p* ≤ 0.05. *Vehicle vs NILO/LPV/IMA; ^†^NILO4µM vs NILO4µM + TEL5µM (in vitro). Mean adiponectin levels in each patient group at different time points were compared against the baseline value. NILO: nilotinib, IMA: imatinib, TEL: telmisartan, LPV: lopinavir

Next, we investigated whether TKIs affect adiponectin in vitro and in plasma samples obtained from CML patients. Adiponectin is a protein exclusively secreted by the adipocyte and is a key mediator of systemic insulin sensitivity and glucose homeostasis [6]. Total adiponectin in the conditioned media collected from drug-treated and control adipocytes were measured using a standard ELISA. Nilotinib induced a dose-dependent reduction in adiponectin secretion (4 µM: 20% reduction, *p* = 0.02); however, the effect of imatinib was only marginal (9.9% reduction, *p* = 0.04). Interestingly, co-incubation of telmisartan with nilotinib reversed the inhibitory effect of nilotinib on adiponectin secretion in vitro (*p* = 0.001; Fig. 2A).

For the in vivo analysis of adiponectin, nonfasted plasma samples at three different time points (baseline, 3 and 12 months) were obtained from 30 CML patients who received either nilotinib (*n* = 14) or imatinib (*n* = 16) for at least 12 months. Relevant ethics approval and patient consent were obtained. All patients were in first chronic phase throughout. In the nilotinib-treated group, six patients received the drug as first-line therapy and eight as second line following initial treatment with imatinib. Five out of the eight second-line nilotinib patients were imatinib-resistant and showed higher *BCR-ABL1* transcript levels at the time of the switch; the remaining three were switched due to imatinib intolerance. In all second-line nilotinib patients, the sample collected at the time of initiation of nilotinib therapy was considered as the baseline sample. All patients in the imatinib-treated group received the drug as first-line. We did not have baseline sample for one of the imatinib-treated patients, therefore we excluded that patient from any analysis (i.e. imatinib, final *n* = 15). The median ages of imatinib and nilotinib-treated CML patients were 39 and 49 years, respectively; both drug groups had eight female subjects each. None of the patients recruited had a medical history of diabetes. Total adiponectin was measured using an electrochemiluminiscence-based sandwich immunoassay (Meso Scale Discovery, USA). Repeated measures ANOVA with Dunnett’s Test was used to compare adiponectin levels at different time points. Imatinib resulted in a significant increase in plasma adiponectin levels at 3 (38.4 ± 7.1 mg/l; *p* < 0.01) and 12 month (36.7 ± 7.2 mg/l; *p* < 0.01) time points compared with baseline values (27.3 ± 5.7 mg/l; *p* < 0.05; Fig. 2B). By contrast, in both first-line (Fig. 2C) and second-line (Fig. 2D) nilotinib patients, there was no change in adiponectin concentrations; however, with second-line nilotinib, there was a non-significant decrease at both 3 (15.2 ± 1.8 mg/l; *p* = NS) and 12 months (14.6 ± 1.7 mg/l; *p* = NS; Fig. 2D) when compared to baseline levels (21.3 mg/l).

Nilotinib-induced reduction in adiponectin in vitro was, to a certain extent, mirrored in the CML plasma samples obtained from second-line nilotinib-treated CML patients, but this was non-significant. However, it should be noted that our sample size was small (*n* = 14 or 15 per drug group) and therefore lacked sufficient power to detect a statistically significant difference in adiponectin. On the other hand, the increase in plasma adiponectin observed with imatinib correlates with what has been previously reported for imatinib in CML patients [14]. Adiponectin expression is directly regulated by PPARγ [6]; it is possible that nilotinib-induced reduction in adiponectin could be a direct result of the downregulation of *PPARγ* by nilotinib. The molecular mechanism(s) by which imatinib increases adiponectin secretion is not clear; it is also possible that the increase in adiponectin levels observed with imatinib in vivo could be a mere reflection of improvement in general health in CML patients.

Here we have shown that repeated exposure of nilotinib and imatinib has contrasting effects on adipocyte lipid accumulation, adipogenic mRNA expression and secretion of adiponectin. Together, these mechanisms may explain the impaired glucose and lipid metabolism observed in nilotinib-treated CML patients. Although aggressive screening for cardiovascular risk factors and cardiometabolic surveillance in CML patients has been suggested to reduce nilotinib-related cardiometabolic events [15], there is also a need for therapeutic preventive strategies. The reversal of nilotinib-induced adipocyte toxicity by telmisartan in vitro is important in this context. The metabolic beneficial effects of telmisartan have been suggested to be due to both PPARγ agonism [8] and angiotensin receptor blockade; the potential therapeutic utility of telmisartan to counter the deleterious cardiometabolic adverse effects caused by nilotinib in CML patients will now need to be evaluated by observational, as well as randomised studies. Our in vivo study has some major limitations, such as small sample size, non-availability of fasting plasma samples and lack of complete concurrent clinical data; future studies will need to address these limitations and validate these results to obtain a better understanding of nilotinib-induced metabolic adverse effects.

## Supplementary information

Supplementary Material

Supplementary Figure 1

Supplementary figure 2
