# Supplementary material for: Nilotinib-induced metabolic dysfunction: insights from a translational study using in vitro adipocyte models and patient cohorts
Source: Leukemia. 2019 Jan 28;33(7):1810–4. doi: 10.1038/s41375-018-0337-0 (PMC6755958; doi:10.1038/s41375-018-0337-0)
Supplement: Supplementary file 1 — Supplementary Material [file 41375_2018_337_MOESM1_ESM.docx]

**Supplementary Material**

1. Materials and methods
2. Supplementary Figures legends
3. Supplementary Bibliography
4. **Materials and Methods**

**Materials**

The 3T3-F442A murine preadipocyte cell line was available in-house in the Wolfson centre for Personalised Medicine, University of Liverpool, UK. Telmisartan was purchased from Sigma-Aldrich (St Louis, MO, USA) while all the remaining drugs (nilotinib, imatinib and lopinavir) were purchased from Santa Cruz Biotechnology (Dallas, Texas, USA). Dimethyl sulfoxide (DMSO), trypsin, dimethylformamide, 3-(4,5-Dimethylthiazol-2-yl)-2,5-Diphenyltetrazolium Bromide (MTT), insulin from bovine pancreas, radioimmunoprecipitation (RIPA) lysis buffer, sodium dodecyl sulphate (SDS), chloroform and Oil Red O were purchased from Sigma-Aldrich. Fetal bovine serum (FBS), Dulbecco’s Modified Eagle’s Medium (DMEM), Hank’s Balanced Salt Solution (HBSS), rat tail collagen I, Reverse transcription kit, TRI Reagent solution, TaqMan gene expression assays (murine) for Peroxisome proliferator-activated receptor-γ (*Pparγ)*, Lipin1 (*Lpin1)*, Sterol regulatory element-binding protein 1 (*Srebp1)*, Glucose transporter 4 (*Glut4)*, Hypoxanthinephosphoribosyltransferase *(Hprt)*, TaqMan gene expression master mix and isopropanol were purchased from Thermo Fisher Scientific (Carlsbad, California, USA). ELISA kits for the estimation of murine adiponectin were obtained from R & D Systems (Minneapolis, MN, USA); electrochemiluminiscence-based immunoassays for the estimation of human adiponectin were obtained from Meso Scale Discovery (Rockville, Maryland, USA).

**Methods**

**Cell Culture**

A chronic *in vitro* toxicity model as previously described^1^ was used to investigate the effect of nilotinib and imatinib on adipocytes. Briefly, 3T3-F442A murine cells were cultured in DMEM containing 10% FBS. The cells were differentiated into adipocytes by the addition of insulin (10mg/ml) for 48 hours. Differentiating adipocytes were incubated with either nilotinib (with or without telmisartan) or imatinib or lopinavir 48 hours post initiation of differentiation. Drug treatment was then carried out every 48 hours over a period of 10 days to mimic the chronic dosing schedule in CML patients. We tested 3 different doses of nilotinib: 1 and 4μM represent the therapeutic range for nilotinib whereas 20μM represents a hypothetical higher nilotinib concentration that may accumulate within the adipocyte following chronic drug treatment. We also assessed the effect of imatinib (5μM; therapeutic concentration) and coincubation of telmisartan (5μM) with nilotinib on the differentiating adipocytes. The concentration of 5μM was selected for telmisartan based on our previous results^1^. Lopinavir (20μM), an antiretroviral drug known to cause adipocyte toxicity and metabolic disturbances^2, 3^, was used as a positive control.

**Measurement of cell viability**

The cytotoxic profile of nilotinib and imatinib in 3T3-F442A preadipocytes and differentiating adipocytes were assessed by the MTT assay. Briefly, both undifferentiated 3T3-F442A cells and differentiating adipocytes were incubated with serial concentrations (0.01 – 100µM) of nilotinib or imatinib for 4 days. On day 4, cells were incubated for 2 hours with MTT and absorbance of the resultant formazan product was measured at 595nm using a multimode detector (Beckman Coulter, Indianapolis, IN, USA).

**Adipocyte lipid accumulation**

Lipid accumulation in differentiated adipocytes was assessed on day 10 (i.e. 48 hours after last addition of drug) using Oil Red O staining as previously described^4^. Briefly, cells were washed with HBSS, fixed with 10% formaldehyde and incubated with Oil Red O solution for 1 hour after which lipid droplet staining was assessed by microscopy. The lipid bound dye was extracted using 70% isopropyl alcohol and absorbance was measured at 520nm. Drug treated cells were compared against vehicle control (methanol).

**RNA extraction and gene expression**

48 hours after final drug addition, total RNA was isolated using the RNeasy kit (Qiagen, Manchester, UK). Total RNA was reverse transcribed using the Taqman® reverse transcription kit (Life Technologies, Paisley, UK). Gene expression of *Pparγ*, *Lpin1, Srebp1*, and *Glut4* were assessed by Real-time PCR using Taqman Assays-on-demand gene expression assays (Life Technologies) on a 7900HT Fast Real Time PCR system (Life Technologies). Hypoxanthinephosphoribosyltransferase *(Hprt)* was used as an endogenous control. The mRNA expression was calculated using the comparative Ct method according to the manufacturer’s protocol and the fold change for the gene of interest was expressed as 2^^-(∆∆CT)^.

**Collection of CML Patient samples**

Nonfasted plasma samples at 3 different time points (baseline, 3 and 12 months) were collected from 30 CML patients who received either nilotinib (n=14) or imatinib (n=16) for at least 12 months. The study was approved by the Liverpool Central (North West 2) Research Ethics committee, and all patients consented to participate in the study. All patients were in first chronic phase throughout. In the nilotinib-treated group, 6 patients received the drug as first line therapy and 8 as second line following initial treatment with imatinib. Five out of the 8 second-line nilotinib patients were imatinib-resistant and showed higher *BCR-ABL1* transcript levels at the time of the switch; the remaining 3 were switched due to imatinib intolerance. In all second-line nilotinib patients, the sample collected at the time of initiation of nilotinib therapy was considered as the baseline sample. All patients in the imatinib-treated group received the drug as first line. We did not have baseline sample for one of the imatinib-treated patients; therefore we excluded that patient from any analysis (i.e. imatinib, final n = 15). None of the patients recruited had a medical history of diabetes.

**Assessment of secreted adiponectin levels *in vitro* and in CML patient samples**

Forty-eight hours post final drug addition, conditioned media were collected from all *in vitro* samples and total adiponectin was measured using ELISA according to the manufacturer’s protocol. Briefly, 100μl of standards and samples were added to the wells of an adiponectin ELISA plate and incubated for one hour with mixing. Secondary antibody was added followed by 1x detector solution. Substrate solution was added followed by incubation in the dark for 20 min at room temperature. Finally, a stop solution was added to stop the reaction and the absorbance was read using a microplate spectrophotometer at 450 nm.

Total adiponectin was also measured in the nonfasted plasma samples collected from CML patients; this was performed by an electrochemiluminiscence-based sandwich immunoassay (Meso Scale Discovery, USA) according to the manufacturer’s protocol. Briefly, antibody-coated plates were incubated with a blocker solution for 1 hour, washed and incubated with 10μl of the sample (1:1000 dilution) or serial dilutions of the calibrator (for preparing the standard curve) for another 2 hours with vigorous mixing. After washing, a detector antibody labelled with SULFO-TAG was added to the wells and incubated for one hour and washed. A Read Buffer was added to the wells and the plates were analysed on the MSD Sector Imager 2400A.

**Statistical Analysis**

All *in vitro* experiments were repeated three times in triplicate. Statistical analyses for *in vitro* studies were conducted by One-way ANOVA with Dunnett’s Test. Repeated measures ANOVA with Dunnett’s Test was used to compare adiponectin levels at different time points in the clinical samples. A p value ≤0.05 was considered significant.

1. **Supplementary figure legends**

**Supplementary Figure 1. Percentage cell viability on incubation with serial concentrations (0.01-100 µM) of nilotinib and imatinib in 3T3-F442A undifferentiated preadipocytes (Fig 1A) and differentiated adipocytes (Fig1B).** All experiments were repeated three times in triplicate. NILO: Nilotinib; IMA: Imatinib.

**Supplementary Figure 2. Effect of nilotinib (with or without telmisartan) and imatinib on adipocyte lipid accumulation in differentiating 3T3-F442A adipocytes.** Lipid droplets were stained with Oil Red O (shown above by red staining) on day 10 following treatment with respective drugs/vehicle and photographed. Telmisartan was coincubated with only one concentration of nilotinib (4µM). Lopinavir (LPV), an anti-HIV drug, was used as a positive control. All experiments were repeated three times in triplicate. NILO: nilotinib; IMA: imatinib; TEL: telmisartan; LPV: lopinavir.

1. **Supplementary Bibliography**

1. Pushpakom SP, Adaikalakoteswari A, Owen A, Back DJ, Tripathi G, Kumar S*, et al.* Telmisartan reverses antiretroviral-induced adipocyte toxicity and insulin resistance in vitro. *Diab Vasc Dis Res.* 2018; 1479164118757924.

2. Djedaini M, Peraldi P, Drici MD, Darini C, Saint-Marc P, Dani C*, et al.* Lopinavir co-induces insulin resistance and ER stress in human adipocytes. *Biochem Biophys Res Commun.* 2009; 386: 96-100.

3. Lagathu C, Bastard JP, Auclair M, Maachi M, Kornprobst M, Capeau J*, et al.* Antiretroviral drugs with adverse effects on adipocyte lipid metabolism and survival alter the expression and secretion of proinflammatory cytokines and adiponectin in vitro. *Antivir Ther.* 2004; 9: 911-920.

4. Boccara F, Auclair M, Cohen A, Lefevre C, Prot M, Bastard JP*, et al.* HIV protease inhibitors activate the adipocyte renin angiotensin system. *Antivir Ther.* 2010; 15: 363-375.
