# Supplementary material for: Nilotinib-induced metabolic dysfunction: insights from a translational study using in vitro adipocyte models and patient cohorts
Source: Leukemia. 2019 Jan 28;33(7):1810–4. doi: 10.1038/s41375-018-0337-0 (PMC6755958; doi:10.1038/s41375-018-0337-0)
Supplement: Supplementary file 2 — Supplementary Figure 1 [file 41375_2018_337_MOESM2_ESM.pptx]

## Slide 1
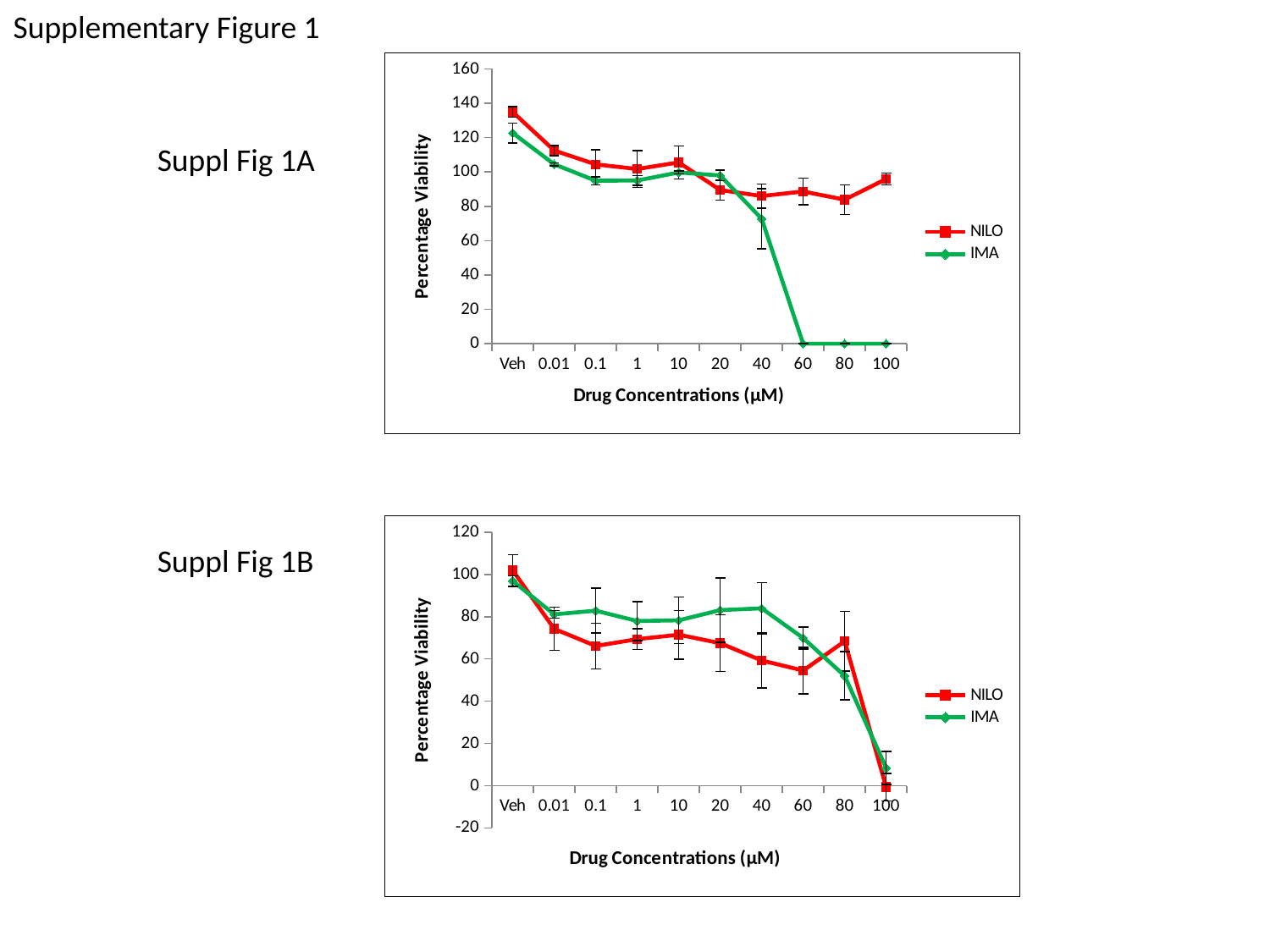

Supplementary Figure 1
### Chart
| Category | NILO | IMA |
|---|---|---|
| Veh | 134.91634839119254 | 122.70489745298418 |
| 0.01 | 112.5224900912433 | 104.49653006924162 |
| 0.1 | 104.41768685244001 | 94.91011873564709 |
| 1 | 101.72324905842454 | 95.01985141375275 |
| 10 | 105.55971530939883 | 99.71436708642636 |
| 20 | 89.51141284839696 | 97.97272386452205 |
| 40 | 85.97225093927534 | 72.76433929103354 |
| 60 | 88.62102345769561 | 0.0 |
| 80 | 83.89255247345177 | 0.0 |
| 100 | 95.91165330980733 | 0.0 |Suppl Fig 1A
### Chart
| Category | NILO | IMA |
|---|---|---|
| Veh | 101.86596128025883 | 96.88652040514432 |
| 0.01 | 74.23961250268161 | 81.09828477497409 |
| 0.1 | 66.14101031668004 | 82.85331559050822 |
| 1 | 69.3633078192459 | 77.94171496655191 |
| 10 | 71.46799384316115 | 78.2825560573655 |
| 20 | 67.48499009038069 | 83.10991179492602 |
| 40 | 59.26938611819704 | 83.96419644353676 |
| 60 | 54.52444071775355 | 69.89416185417475 |
| 80 | 68.36535839728278 | 51.99133317618086 |
| 100 | -0.6759464663442474 | 8.350879483198353 |Suppl Fig 1B
