# Supplementary figures and images for: Nilotinib-induced metabolic dysfunction: insights from a translational study using in vitro adipocyte models and patient cohorts
Source: Leukemia. 2019 Jan 28;33(7):1810–4. doi: 10.1038/s41375-018-0337-0 (PMC6755958; doi:10.1038/s41375-018-0337-0)

## Slide 1
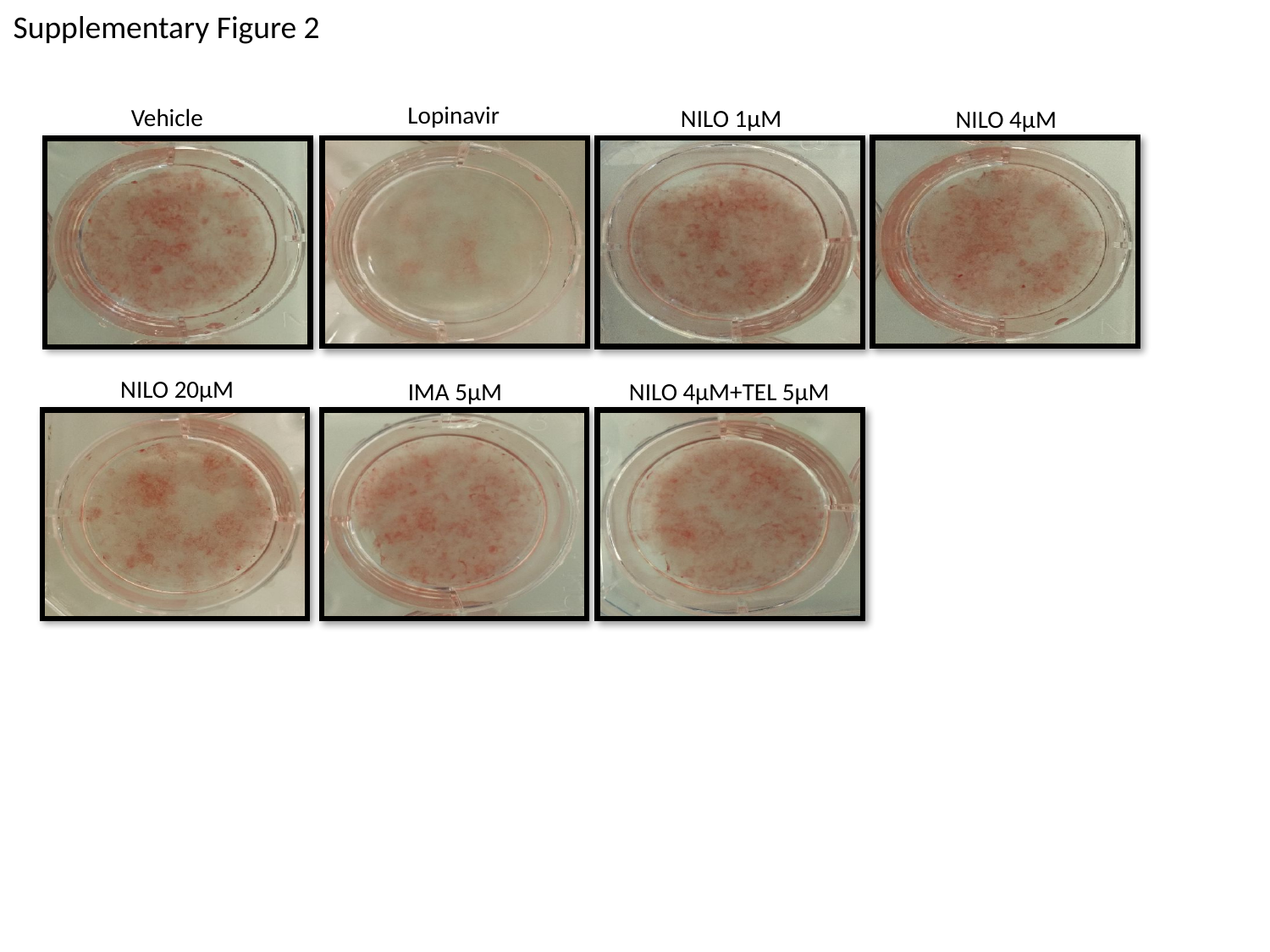

Supplementary Figure 2
Lopinavir
Vehicle
NILO 1µM
NILO 4µM
NILO 20µM
IMA 5µM
NILO 4µM+TEL 5µM

Supplement: Supplementary file 3 — Supplementary figure 2 [file 41375_2018_337_MOESM3_ESM.pptx]
